# The First Rapid Assessment of Avoidable Blindness (RAAB) in Thailand

**DOI:** 10.1371/journal.pone.0114245

**Published:** 2014-12-11

**Authors:** Saichin Isipradit, Maytinee Sirimaharaj, Puwat Charukamnoetkanok, Oraorn Thonginnetra, Warapat Wongsawad, Busaba Sathornsumetee, Sudawadee Somboonthanakij, Piriya Soomsawasdi, Umapond Jitawatanarat, Wongsiri Taweebanjongsin, Eakkachai Arayangkoon, Punyawee Arame, Chinsuchee Kobkoonthon, Pannet Pangputhipong

**Affiliations:** Mettapracharak (Wat Rai Khing) Hospital, 52 Moo 2 Rai Khing, Sampran, Nakornprathom 73210, Thailand; Medical University of South Carolina, United States of America

## Abstract

**Background:**

The majority of vision loss is preventable or treatable. Population surveys are crucial for planning, implementation, and monitoring policies and interventions to eliminate avoidable blindness and visual impairments. This is the first rapid assessment of avoidable blindness (RAAB) study in Thailand.

**Methods:**

A cross-sectional study of a population in Thailand age 50 years old or over aimed to assess the prevalence and causes of blindness and visual impairments. Using the Thailand National Census 2010 as the sampling frame, a stratified four-stage cluster sampling based on a probability proportional to size was conducted in 176 enumeration areas from 11 provinces. Participants received comprehensive eye examination by ophthalmologists.

**Results:**

The age and sex adjusted prevalence of blindness (presenting visual acuity (VA) <20/400), severe visual impairment (VA <20/200 but ≥20/400), and moderate visual impairment (VA <20/70 but ≥20/200) were 0.6% (95% CI: 0.5–0.8), 1.3% (95% CI: 1.0–1.6), 12.6% (95% CI: 10.8–14.5). There was no significant difference among the four regions of Thailand. Cataract was the main cause of vision loss accounted for 69.7% of blindness. Cataract surgical coverage in persons was 95.1% for cut off VA of 20/400. Refractive errors, diabetic retinopathy, glaucoma, and corneal opacities were responsible for 6.0%, 5.1%, 4.0%, and 2.0% of blindness respectively.

**Conclusion:**

Thailand is on track to achieve the goal of VISION 2020. However, there is still much room for improvement. Policy refinements and innovative interventions are recommended to alleviate blindness and visual impairments especially regarding the backlog of blinding cataract, management of non-communicative, chronic, age-related eye diseases such as glaucoma, age-related macular degeneration, and diabetic retinopathy, prevention of childhood blindness, and establishment of a robust eye health information system.

## Introduction

Blindness and visual impairment contribute to the disability-adjusted life year (DALY)— a measure of overall disease burden, expressed as the number of years lost due to ill-health, disability or early death. One DALY can be thought of as one lost year of "healthy" life. The inability to see well profoundly reduces quality of life and has tremendous economic impacts [1]–[3]. While care and rehabilitation provided to the blind and visually impaired are the most obvious costs, equally significant but less apparent are the indirect costs resulting from the loss of productivity of the affected persons and their loved ones.

The majority of blindness is preventable or treatable [4]. As evidenced by millions of people who undergo cataract surgery to cure this major sight robbing disease, the simple act of restoring sight not only enhances productivity, but also returns mental and spiritual richness to one's life. Thus, inaction in the face of preventable blindness is morally and economically indefensible. However, in many parts of the world, too many people with cataract still needlessly suffer from this leading treatable ocular disease.

VISION 2020 is a global movement to eliminate avoidable blindness by the year 2020. Elimination refers to reducing the conditions to the extent that they are no longer public health problems. Thus the aspiration of VISION 2020 is to bring avoidable visual impairments down to a level with which can be coped by the regular health system, without recourse to campaign approaches or outreach case-finding and treatments [5]. In many countries, this goal requires a systemic reform that engages all relevant stakeholders to strike an optimal balance among sustainable infrastructure, appropriate technologies, and skillful human resources.

Evidence from the population is crucial to plan, implement, and evaluate actions necessary to realize this worthy goal [6]. Currently, reliable metrics of progress or measures of success can only come from population survey [7]–[9]. This is a report of the first Rapid Assessment of Avoidable Blindness (RAAB) Survey in Thailand conducted to estimate the prevalence of blindness and visual impairment and to identify under-serviced ocular diseases.

## Methods

### Sample Size Calculation

A prevalence of blindness of 0.59% in people aged 50 and above was assumed, based on experience from the 4th National Survey of Blindness and Visual Impairment in Thailand. Using a 95% confidence interval, a precision of 0.5% (worst acceptable prevalence 1%), design effect (DEFF) of 1.6 and a non-response rate of 5%, the calculated sample size was 21,000.

### Sampling Method

This survey used stratified four-stage cluster sampling. Using the Thailand National Census 2010 as the sampling frame, we selected the subjects using a probability proportional to size systematic sampling in which provinces, districts (Ampoe), subdistricts (Tumbon), and Enumeration Areas (EAs) were the primary, secondary, tertiary, and quaternary sampling selection strata respectively (Table 1). The data collection unit was the Thai population 50 years or older residing in the EA.

**Table 1:** Details of sampled provinces and enumeration areas (EAs) in each regional stratum.

Strata	Numbers of Sampled Provinces	Names of Sampled Provinces	Numbers of Sampled EAs
Bangkok	1	Bangkok	16
Central (except Bangkok)	3	Kanjanaburi, chantaburi, Patumtani	48
North	2	Chiang Mai, Petchaboon	32
Northeast	3	Nakornrajsima, Khon Kaen, Yasothorn	48
South	2	Chumporn, Songkra	32
Total	11		176

A list of provinces organized geographically according to Regions (Phak) provided the framework for primary sampling selection. Within each regional stratum, the provinces were selected using simple random sampling. This resulted in 11 provinces distributed throughout each stratum. The framework for secondary sampling selection was a list of districts in each sampled province. Within each sampled province, two districts were randomly and independently selected according to a probability proportional to size systematic sampling. A total of 22 districts were chosen. In the next stage, tertiary sampling selection was based on a list of subdistricts in each sampled district. For each sampled district, two subdistricts were randomly and independently selected according to a probability proportional to size systematic sampling. A total of 44 subdistricts were chosen. The fourth stage of stratified sampling selection used a list of EAs in each sampled subdistrict organized by demarcation pattern from the Department of Provincial Administration. Within each sampled subdistrict, four EAs were randomly and independently selected according to probability proportional to size systematic sampling. A total of 176 EAs were chosen. Samples of population age 50 years or older were selected from these EAs using systematic cluster random sampling. The targeted number of sampled individuals was 21,000.

### Ophthalmic Evaluation and Data Collection

In each area, the survey team set up a temporary examination center where all the examinations were conducted between August 2012 and July 2013. The local community health volunteers along with the Public Relations Officer of the team briefed the people in the selected cluster about the survey in advance. The eligible subjects in each household were accompanied to the examination center by local health volunteers to facilitate compliance. Due to relatively common illiteracy in many survey areas, oral informed consent was taken from each eligible participant. If an eligible person was not available during the survey, at least two more attempts were made to assess information. If after repeated visits, examination could not be done, information of his or her visual status was obtained from relatives or neighbors.

The RAAB standardized set of questions, translated into Thai, was completed for each eligible person. They were divided into seven parts including: general demographic information; whether known to have diabetes mellitus; visual acuity and lens assessment; principal cause of visual impairment; reasons why cataract surgery had not been done; and information about cataract surgery done including time, place and level of patient satisfaction.

Visual acuity (VA) was measured using a tumbling ‘E’ chart, which had an Snellen optotype size 18 on one side and 60 on the other side at a 6 or 3 meters distance, and was measured in full daylight with available correction. Pinhole VA was measured when the VA was <20/70 in either eye. The lens was examined in both eyes using a direct ophthalmoscope or slit-lamp and was graded according to the following categories; ‘normal lens’, ‘obvious lens opacity’, ‘lens absent (aphakia)’ or ‘IOL implantation’. If corneal opacity precluded lens examination, then the examiner recorded ‘no view of lens’.

If the VA improved to 20/70 or better with pinhole, then refractive error was assigned as the cause of visual impairment. If it was found that the vision in either eye was <20/70 with a pinhole, a more detailed examination was performed of the anterior segment to elicit the cause. If the cause was not in the anterior segment, the posterior pole was examined by non-mydriatric fundus camera. If the cause was still not evident, the ophthalmologist proceeded to dilate the pupil and perform fundoscopic examination. Cataract was assessed according to the crystalline lens opacity. Glaucoma was defined as the principal cause of visual impairment if the optic cup-to-disc ratio was greater than 0.6, in the absence of another cause of reduced vision.

The principal cause of visual impairment was based on the WHO convention whereby the principal cause is attributed to the primary disorder. If there are multiple causes, then the cause that can be most easily treated was assigned as the principal cause.

All patients with eye problems requiring further medical attention received appropriate referral. The survey team also made certain to clearly communicate these and other relevant information to the patients and local health personnel.

### Data entry and Analysis

Four clerks were trained in data entry and double entered the data from the survey. The RAAB software allowed assessment of data entry errors and missing information.

### Categories of Visual Impairment

Three WHO categories for visual impairment, based on presenting visual acuity (VA) with available correction in the better eye, are defined as follows: blindness, VA <20/400 in the better eye; severe visual impairment, VA <20/200 but ≥20/400 in the better eye; and moderate visual impairment, VA <20/70 but ≥20/200.

### Statistical Analysis

The survey software ‘RAAB version 5.0 (Health Information Services and Tax Software) was used. This software allows data entry, sample size calculation, and standardized data analysis. The prevalence estimates considered the design effect when estimating the confidence intervals because cluster sample was used rather than simple random sampling. Estimations of the number of cases with blindness, severe visual impairment and moderate visual impairment were obtained by extrapolating the age-sex-specific prevalence estimates to the age-sex structure of the population.

### Ethical Approval

The Mettapracharak (Wat Rai Khing) Hospital Ethics Committee granted approval for this study. The research followed the tenets of the Declaration of Helsinki. To address the issue of illiteracy, verbal informed consent was obtained from each person included in the study and noted in the record form. The Ethics Committee approved this consent procedure.

## Results

A total of 21,000 persons over the age of 50 years old were enumerated and 20,044 (95.4%) were examined in eleven of Thailand's provinces. Table 2 showed the composition of population, in sample and in survey area, by age and sex.

**Table 2:** Composition of population in sample and in survey area, by age and sex.

Age Group/Sexes	Survey Areas		Examined Sample	
	(N)	%	(n)	%
50–59 years	8,014,911	49.5	8,643	43.1
60–69 years	4,541,021	28.1	7,523	37.5
70–79 years	2,542,247	15.7	3,245	16.2
80+ years	1,087,641	6.7	633	3.2
Male	7,422,027	45.9	7,455	37.2
Female	8,763,793	54.1	12,589	62.8
Total	16,185,820		20,044	

Among the surveyed population, the age and sex adjusted prevalence of blindness (vision <20/400 in the better eye with available correction) was 0.6% (95% CI: 0.5–0.8), severe visual impairment (SVI) (<20/200–20/400 in the better eye with available correction) was 1.3% (95% CI: 1.0–1.6), and moderate visual impairment (MVI, <20/70–20/200 in the better eye with available correction) was 12.6% (95% CI: 10.8–14.5). The prevalence of MVI, SVI, and blindness increased with age. In this survey, women had a similar prevalence of blindness but a higher prevalence of MVI and SVI compared to men (Table 3).

**Table 3:** Distribution of visual acuity with available correction in the better eye.

Presenting Visual Acuity	Male		Female		Total	
	N	Prevalence (95% CI)	N	Prevalence (95% CI)	N	Prevalence (95% CI)
Blindness	50,008	0.7% (0.5–0.9)	50,524	0.6% (0.4–0.7)	100,532	0.6% (0.5–0.8)
Severe Visual Impairment	82,072	1.1% (0.8–1.4)	130,191	1.5% (1.2–1.8)	212,263	1.3% (1.0–1.6)
Moderate Visual Impairment	852,625	11.5% (9.4–13.5)	191,576	13.6% (11.7–15.5)	1,044,201	12.6% (10.8–14.5)

Using direct standardization, regional analysis of the age and sex adjusted prevalence of blindness, SVI, and MVI in Thailand is shown in Table 4. Each region demonstrated similar adjusted prevalence except the Bangkok area, which appeared to have lower prevalence of blindness.

**Table 4:** Age and Sex Adjusted Prevalence of Blindness, SVI, and MVI in each region of Thailand.

Presenting Visual Acuity	North	Northeast	Central	South	Bangkok	Thailand
	Prevalence (95% CI)	Prevalence (95% CI)	Prevalence (95% CI)	Prevalence (95% CI)	Prevalence (95% CI)	Prevalence (95% CI)
Blindness	0.7% (0.4–0.9)	0.7% (0.5–1.0)	0.6% (0.3–1.0)	0.6% (0.3–0.9)	0.3% (0.1–0.6)	0.6% (0.5–0.8)
Severe Visual Impairment	1.5% (0.8–2.2)	1.8% (1.3–2.3)	1.1% (0.7–1.5)	0.9% (0.4–1.5)	0.6% (0.0–1.3)	1.3% (1.0–1.6)
Moderate Visual Impairment	11.0% (7.0–15.4)	14.6% (10.7–18.6)	11.1% (8.4–13.9)	11.2% (7.0–15.4)	15.7% (8.6–22.9)	12.6% (10.8–14.5)

Cataract remained the most common cause of overall blindness and avoidable blindness and VI (Tables 5,6). Cataract was responsible for 69.7% of blindness. Refractive errors including uncorrected aphakia were responsible for 6.0% of blindness while complications after cataract surgery were responsible for 1% of all blindness. Diabetic retinopathy was responsible for 5.1% of blindness while glaucoma and corneal opacities were responsible for 4.0% and 2.0% of blindness respectively. Uncorrected refractive errors were responsible for almost a third (26.7%) of MVI and were the second most common cause after cataract (60.0%) among those with MVI. Among the blind, 92.1% lost their vision from avoidable causes, while among VI, 97.9% was due to avoidable visual impairment (Table 6).

**Table 5:** Proportion of Blindness, SVI, and MVI in examined persons due to specific causes in Thailand.

Causes	Blindness (n = 99)	SVI (n = 236)	MVI (n = 2,431)
	%	%	%
Cataract untreated	69.7	77.1	60.0
Refractive error	4.0	10.2	26.7
Aphakia uncorrected	2.0	0.0	0.1
Cataract surgical complications	1.0	0.4	0.9
Glaucoma	4.0	3.4	3.2
Diabetic retinopathy	5.1	2.1	1.1
Corneal opacity	2.0	2.1	3.2
Phthisis	4.0	2.5	2.5
AMD	2.0	0.4	0.1
Other posterior segment diseases	6.1	1.7	2.1
All other globe/CNS abnormalities	0.0	0.0	0.0
Onchocerciasis	0.0	0.0	0.0

**Table 6:** Causes of Blindness, SVI, and MVI categorized by possible interventions.

Interventions	Blindness (%)	SVI (%)	MVI (%)
Treatable^1^	76.8	87.3	86.6
Preventable (PHC/PEC services)^2^	6.9	5.3	5.7
Preventable (Ophthalmic services)	10.6	6.4	5.3
Avoidable^3^	92.1	97.9	97.8
Posterior segment causes	20.5	7.6	6.6

1 Refractive error + uncorrected aphakia + untreated cataract

2 PHC  =  Primary Health Care, PEC  =  Primary Eye Care

3 Avoidable  =  Treatable + Preventable

Table 7 displayed the cataract surgical coverage (the percentage of people in the survey who had cataract surgery compared to the number who required it). The cataract surgical coverage (CSC) for blind persons was 95.1% (91.7% for males and 96.5% for females), for SVI persons it was 85.3% (81.6% males, 87.2% females) and for those with MVI it was 46.6% (42.6% males, 48.8% females). The overall CSC for blind eyes was high (79.6%). Despite relatively high cataract surgical coverage, the backlog for blinding cataract (people waiting who might benefit from surgery) was calculated (69.7% of 100,532) to be 70,071 persons. If the cut-off for cataract surgery is set to 20/200 (a cut off for cataract surgery for the universal coverage scheme in Thailand is 20/100) then this study suggests that there are approximately 143,886 people (46.0% of 312,795) in Thailand who might benefit from surgery in at least one eye.

**Table 7:** Cataract surgical coverage in persons and eyes.

Cataract Surgical Coverage	Male %	Female %	Total %
Persons			
VA <3/60	91.7	96.9	95.1
VA <6/60	81.6	87.2	85.3
VA <6/18	42.6	48.8	46.6
Eyes			
VA <3/60	73.4	83.1	79.6
VA <6/60	64.1	72.0	69.2
VA <6/18	29.5	35.1	33.0

## Discussion

Thailand was one of the earliest countries to designate visual impairment as a national health priority and to initiate a primary eye care program for the control of this major global public health challenge [10]. An effort to harness evidences to understand current situation and to discover unmet need is a prerequisite for inspiring support of the blindness prevention program and guiding its implementation. Population based surveys have been the main source providing information to refine the program and ensuring that it was progressing in the right direction [7], [11]–[17]. In Thailand, four national eye surveys— summarized in Table 8— were previously embarked at periodic intervals over the past two decades [18].

**Table 8:** Comparison among five National Eye Surveys in Thailand.

National Eye Survey	Year	Blindness Prevalence	% Cataract	Surgical Backlog
1st	1983	1.14%	47.3%	270,000
2nd	1987	0.58%	71.3%	220,000
3rd	1994	0.31%	74.7%	134,000
4th	2006	0.59%	51.6%	98,336
5th	2013	0.60%	69.7%	70,071

Strengthening the eye health system requires the planning, implementation, monitoring and evaluation of appropriate eye care services. To be effective, these efforts, in turn, need evidence generated from populations. However, a comprehensive national vision survey is costly and involves herculean efforts. Therefore, important evidence, needed for policy planning and evaluation, often has not been adequately and consistently produced.

Rapid assessment techniques have been developed which provide valid estimates in a short period of time and also reduce the overall cost of conducting a survey [19]–[22]. With the call for the elimination of avoidable blindness by the year 2020, rapid assessments have evolved to include all causes of avoidable blindness like cataract, refractive errors, trachoma and other causes of corneal scarring [9]. This study represents the 5^th^ Thailand National Eye Survey (Table 8). To our knowledge, this is the first rapid assessment of avoidable blindness (RAAB) study in Thailand.

In this study, the prevalence of blindness among those aged 50 years and over was 0.6% (95% CI: 0.5–0.8). This is lower than the prevalence reported among many other countries in Southeast Asia [23]–[26]. The WHO suggested that a country as a whole should bring national blindness prevalence rates to less than 0.5% and not more than 1% in any district in the country. This range was recommended in the interests of equity, taking into account unavoidable causes of blindness [27]. Recently, the World Health Assembly adopted resolution WHA66.4 that set a global target, the reduction in prevalence of avoidable visual impairment by 25% by 2019 from the baseline of 2010 [28].

The cataract surgical coverage is very good in every region of Thailand reflecting the success of primary eye care (a strong commitment to integrate basic eye care into the primary health care delivery system within the community) and national programs to prevent blindness and visual impairment. However, the surgical backlog of visually significant cataract remains unacceptably high. This can be explained in two ways. First, the coverage fails to reach significant number of people with blinding cataract. And/or, second, the cataract surgical rate (CSR; number of cataract surgery per a million people per year) is not high enough to absorb new cases of blinding cataract. [29]

Between the 4th Thailand national survey and this one, the National Health Security Office (NHSO)— tasked with administration of universal health coverage (UC)— has provided coverage for cataract surgery both under routine health care and as additional financial incentive for clearing the surgical backlog. While comparing studies with different methodology across time is a complex task, the result of this study seems to indicate that this laudable effort did not seem to significantly lessen the prevalence of blindness and visual impairments. More importantly, the proactive UC cataract scheme has not been adequate to address the surgical backlog of blinding cataract. This policy, providing minimally regulated, semi-free-market-based incentive to clear cataract surgical backlog, needs to be rethought and modified.

The Ministry of Public Health of Thailand is currently introducing an area health initiative called the Service Plan. The idea is to geographically divide the country into 12 health areas plus the Bangkok Metropolitan. Each integrated health area will have the autonomy to manage resources to find the synergy that ensures the health and well being of the area's citizens. The idea is to minimize the need for inter-area referral by having adequate local expertise to deal with most health issues and only reserving the referral system for the very complex cases to be cared for by the centers of excellence.

The Eye Service Plan is among the 11 specialties covered by the first phase of this initiative. One important strategy of the Eye Service Plan is the use of public health volunteers to identify blinding cataract cases within community coupling with effective surgical referral to clear backlog of blinding cataract within the Service Plan's health areas. We will have an opportunity to assess the impact of the Service Plan by comparing the result of this study with the next one about five years from now. The next RAAB survey in Thailand will also provide information on where Thailand stands in terms of the VISION 2020's indicators.

This study provides evidence that Thailand is on track to achieving the goal of VISION 2020. However, there is no time for complacency. We must continue to encourage political and professional commitment to prevention of visual impairment and to elevate these good results with continuous improvement and innovations. Therefore, it is essential to reenergize the National Committee for the Prevention of Blindness and to update Thailand's Vision 2020 Action Plans.

One important trend seen in this study is the rising significance, in Thailand, of chronic, non-communicable, and age related eye diseases such as glaucoma, age-related macular degeneration, and diabetic retinopathy. This global trend requires a fresh approach to screening and chronic eye care [30]. Furthermore, by design, this rapid survey does not give information regarding the eye health of children. Clearly, including children in strategies and programs to prevent blindness and visual impairments is very important because many proven effective interventions are available and there is a huge potential for long lasting future benefits.

Like many developing nations, the eye health system of Thailand is facing a triple burden of ill eye health— the unfinished business of blinding cataract surgical backlog, the emerging time bomb of chronic, age-related eyes diseases, and the future-critical task of preventing blindness in children. To effectively and sustainably address these challenges, we must revitalize and strengthen primary eye care in Thailand. More over, to cultivate this community-based, system-approach to eye care in the upcoming ASEAN Community, we will have the opportunity to renew, re-imagine, and redesign the Korat Institute of Public Health Ophthalmology's role in eye health care personnel training and regional collaboration [31].

Finally, RAAB provides focus and simple methodology to understand blindness and visual impairments. But the fact remains that population surveys are labor intensive and require large resources. Surveys also provide only a snapshot in time of the magnitude, geographic distribution and causes of blindness and visual impairments. Optimal understanding of the diverse, complex, dynamic eye health system requires a robust eye health information platform that is capable of providing real time data. A move from survey toward a comprehensive eye health information system including a mobile-device-enabled [32], web-based surveillance system is a challenging but worthwhile goal.
